# Determinants of Human Coronary Collaterals

**DOI:** 10.2174/1573403X1001140317114411

**Published:** 2014-02

**Authors:** Stefano F. de Marchi

**Affiliations:** Department of Cardiology, University Hospital, Bern, Switzerland

**Keywords:** Coronary circulation, collateral circulation, determinants.

## Abstract

The human coronary collateral circulation is prognostically relevant. The understanding of collateral formation and its determinants may guide future therapeutic strategies aiming at promoting collateral growth and functionality, and hence reducing the global burden of coronary artery disease (CAD).

## INTRODUCTION

The human coronary collateral circulation is prognostically relevant [1]. The understanding of collateral formation and its determinants may guide future therapeutic strategies aiming at promoting collateral growth and functionality, and hence reducing the global burden of coronary artery disease (CAD) [2].

## PREFORMED CORONARY COLLATERALS

The prenatal development of the coronary circulation is marked by a key-event, i.e. the formation of the coronary stems in the aortic root and the onset of flow in the preformed fetal coronary plexus [3, 4]. A layer of mesenchymal cells surrounding the endothelium differentiates into the tunica media, and smooth muscle cells start expressing actin and myosin [5]. Although the coronary vascular growth extends beyond birth [6, 7], the structure simultaneously undergoes a major morphologic change. Starting from an interconnecting tubular mesh grid, the supply routes progressively concentrate on fewer major distribution channels, giving rise to the well-known branching pattern of adult epicardial arteries [8]. Although at a first (angiographic) glance, it may seem that arterio-arterial anastomoses regress completely in humans, it has repeatedly been shown that collateral channels abundantly persist through adult life. Using glass microsphere injections in post-mortem human hearts, Prinzmetal found anastomoses up to 70 to 180 μm in luminal diameter in all examined hearts [9]. In 1963, Fulton showed that the presence of anastomoses is ubiquitous in normal human post-mortem hearts [10]. The diameters were found to reach 500 μm, but were mostly within the range of 40-200 μm. Such small vessels must be considered non-functional due to their excessive resistance to flow and the functional relevance of preformed collaterals has long been a matter of debate. Using quantitative collateral measurements *in vivo*, Wustmann demonstrated that "functional" collaterals (defined as collaterals preventing signs of ischemia during brief coronary occlusions) prevail in one fifth of adult patientswithout stenotic CAD [11]. Furthermore, a large interindividual variability of collateral flow was observed, raising fundamental questions about the functionality of preformed collaterals and their determinants, such as genetic predispositions, environmental factors, and the role of co-morbidities. 

The mechanisms by which these small collaterals remain sufficiently evolved to protect myocardium from ischemia during coronary occlusion are still unclear. It may be assumed that a certain amount of blood flow must persist in order to maintain lumen patency under physiological condition. Unlike during coronary obstruction, where a large interarterial pressure-gradient clearly distributes the roles of donor and receiver vessel, such an obvious driving force is missing in hearts without obstructive CAD. In theory, collateral flow can be generated whenever coronary arteries exhibit pressure changes that are not in perfect synchrony. Since pulse waves travel at different speed in different arteries (depending on luminal diameter and wall properties), pulsatile collateral flow might not only be generated by the physiological asynchrony of cardiac contraction/relaxation, but also on the basis of the anatomical heterogeneity of coronary arteries, particularly in smaller vessels [12-15]. Schaper suggested that in normal hearts, flow might enter the collaterals from all anastomotic ends simultaneously, being drained from branches located at the collateral vessel itself into a capillary system. In such a case, all coronary arteries would be donors, while the collaterals would form an arterial arcade. This view is interesting, because an arcade-like aspect can be guessed in some collaterals shown in Fultons images [16]. A conclusive demonstration of the anatomy of preformed coronary collaterals might only be given by a systematic hierarchical microimaging approach, as previously proposed by Heinzer *et al*. for the cerebral circulation [17]. 

## DETERMINANTS IN EARLY LIFE

Investigations on determinants of preformed coronary collaterals in humans have been ongoing for decades. An early study on this subject was performed in an attempt to investigate the role of genetic predisposition. In 1960, Pepler *et al*. compared the collateral preformation between Europeans and Africans using post-mortem arteriography [18]. Among the 23 normal hearts in the European group, only 8 showed well-developed anastomoses (35%), whereas in the African group, this was true in 26 of 49 hearts (54%). In the conclusions, the authors did not attribute this difference to an "inherited anastomotic blood supply", but rather to the widespread iron-deficiency and megaloblastic anemia in very young African children, leading to a "stimulation of the development of intercoronary arteriolar anastomoses". This study was of particular interest, not necessarily because of its primary scope to determine genetic differences, but rather for pointing on factors modulating collateral preformation in early life. Reiner *et al*. confirmed the role of such factors. They found that almost 4 of 5 neonates had a well-developed collateral circulation (>40 μm), and that there was a natural involution process in the course of childhood, except in children with an apparently pathologic growth stimulus, such as anemia, anoxia and hypertrophy [19]. Furthermore, maternal age <23 years at birth was also found beneficial for collateral preformation. A very interesting group is undoubtedly formed by children with congenital heart disease, because such pathologic stimuli are particularly strong. Bloor *et al*. measured the diameter of intercoronary anastomoses using wax sphere injections in 35 post-mortem hearts with congenital defects (age 0-19) and compared them with 20 hearts without disease (age 0-14) [20]. The collateral diameter was in the range 20-74 μm in all controls, whereas in the congenital group, only 46% were within the same range, but 54% were in a superior range of 20 -120 μm. Thus, the collateral vessels where better developed in the congenital group. Interestingly, the data showed that the collateral size significantly increased with age in the congenital group. The largest collaterals (> 100 μm) were found exclusively in children older than 5 years, indicating that the stimulus requires a long period before becoming effective. A subgroup analysis was additionally performed to study the influence of hypoxemia by comparing cyanotic and acyanotic congenital heart disease, but no significant difference was found. However, the statistical power of this subgroup analysis was low. 

## GENETIC DETERMINANTS IN HEARTS WITHOUT CORONARY ARTERY DISEASE

In the era of modern laboratory technology, gene expression analysis allows assessing the genetic determinants of collateral preformation. An important contribution in this field was provided by Meier *et al*. in 2009 [21]. In 160 adult patients with and without CAD, microarray gene chip and polymerase chain reaction (PCR) were used to identify marker genes in isolated RNA of peripheral blood monocytes. These cells are known to be of fundamental importance for arteriogenesis, and hence for collateral growth [22, 23]. Collateral function was determined quantitatively by the pressure-derived collateral flow index (CFI) [24]. Fifty of the included patients had no coronary lesions in angiography or fractional flow reserve abnormalities [25-27]. This subgroup is of particular interest, because it allows observing gene expression in patients with various degrees of collateral preformation, but without the strong growth stimuli typical for obstructive CAD. After applying restrictive selection criteria, 13 marker gene expressions were found to correlate significantly with CFI. Among these, 3 were also significant predictors of sufficient collateralization (i.e. CFI ≥ 0.21) in receiver-operator characteristic (ROC) analysis, namely: ACTN1 (integrin pathway), FGF13 (angiogenesis pathway), and RPS6KA3 (platelet-derived growth factor pathway). 

## HEARTS WITHOUT CORONARY ARTERY DISEASE: CLINICAL DETERMINANTS IN ADULTHOOD

In a large series of 1271 healthy and diseased post-mortem human hearts, Zoll *et al*. performed a comprehensive analysis of collateral (pre-)formation and its determinants [28]. Among the dissected hearts, 244 were considered normal by conservative exclusion criteria. Intercoronary anastomoses were assessed using injections of colored lead-agar mass, a tracer that penetrates vessels as small as 40 μm. Abundant collaterals were found in 55 hearts (23%). In 101 non-anemic individuals, only 9 hearts (9%) showed such collaterals, whereas in 91 persons who suffered from anemia, they were found in 35 hearts (39%). In the remaining 51 cases, no classification into a specific anemia group was possible. Since well-developed collaterals were found in 11 of 15 hearts with cor pulmonale, the authors concluded that the underlying myocardial hypoxia was responsible for stimulating anastomoses, and that heterogeneous regional hypoxia patterns may have provoked pressure gradients driving flow in preformed collaterals. Regional differences in susceptibility to ischemia have recently been demonstrated [29]. 

In 2011, our group published the first analysis of collateral determinants in 106 patients without CAD using quantitative in-vivo collateral measurements [30]. In this study, 39 clinical variables were screened for their potential as independent determinant of CFI. These included various hemodynamic parameters, laboratory findings, co-morbidities, general characteristics and cardiovascular drugs. In univariate analysis, age, height, arterial pulse pressure amplitude, LV end-diastolic pressure (LVEDP), heart rate, arterial hypertension, dyslipidemia, statins and diuretics fulfilled the initial selection criterion. In a subsequent multiple regression analysis, LVEDP (direct), heart rate (particularly in patients without betablockers; inverse) and hypertension (inverse) were independently related to CFI, explaining 30% of the observed variability. Although some of the unexplained variation is caused by measurement inaccuracies, more determinants of collateral preformation can be expected, such as gene expression [21], stimuli in childhood [19], and unknown factors. LVEDP was considered a false indicator of increased CFI because at high values, a pressure-derived flow index no longer relates to flow due to a collapse of the microcirculation, leading to high positive zero-flow pressure (waterfall mechanism) [31-33]. More interest is drawn by the two remaining determinants: low heart rate and absence of arterial hypertension. A potential collateral-stimulating role of bradycardia has previously been reported in 61 patients with CAD using angiographic collateral grading [34]. In bradycardia, the diastole-to-cycle duration ratio increases, thus augmenting the overall time at which coronary flow is not impeded by systolic microvascular compression. Since shear stress is the major determinant for arteriogenesis, lowering heart rate was expected an attractive means to stimulate collateral growth. From the multiple regression equation, it could be predicted that lowering heart rate from 80 to 60 cycles per minute would be associated with an average increase of CFI from 0.18 to 0.26, thus from an insufficient level to a value above the protective threshold [30, 35]. A randomized, heart rate lowering trial of our group using ivabradine will elucidate the effect on collateral function. Regarding the other important determinant of CFI, i.e. absence of hypertension, the results were very consistent in the statistical analysis. Only one of two previous studies on the role hypertensive heart disease has shown similar results, but both used a crude angiographic estimation of collateralization [36, 37]. It can be speculated that functional and structural changes associated with hypertension (i.e. medial thickening [38], rarefaction of the microcirculation [39], vasomotor dysfunctions [40], delayed relaxation [41] are detrimental to shear-stress-induced vessel growth. 

It is noteworthy, however, that some variables that were expected to play a role in collateral preformation were found unrelated to CFI, such as smoking, diabetes, and hemoglobin levels. In particular, the lack of any relation between hemoglobin and CFI was surprising, as it is in overt contradiction to the early study of Zoll [28]. It must be noted, however, that the severity and duration of anemia in our study population is certainly lower, because anemia is treated more vigorously today compared to 60 years ago. Furthermore, hemoglobin levels tend to vary over time and reductions from single measurements may not represent a prolonged anemic state. Diabetes and smoking, both are conditions affecting the coronary macro- and microcirculation, were far from any statistical relation to CFI. These results were consistent with other investigations [42]. More doubts arise with respect to age and dyslipidemia, both initially selected as candidates in univariate, but then rejected in multivariate testing. For age there was a trend toward an inverse relation to CFI, whereas a clear relation with dyslipidemia might have been blurred by the high prevalence and effectiveness of lipid-lowering treatment.

## CORONARY ARTERY DISEASE: STENOSIS SEVERITY AND BEYOND

There is no doubt that the most potent stimuli for collateral growth are triggered in obstructive CAD. The existence and relevance of collaterals have long been debated. After 1883, when West published several autopsy cases in which total coronary occlusion had not been fatal [43], a century of post-mortem studies involving innumerable detection and measurement approaches has revealed the importance of coronary narrowing as a trigger for collateral formation. In the previously mentioned post-mortem series of Zoll *et al*., the effect of incremental stenosis severity on collateral formation was demonstrated [28]. While according to their (limited) method, anastomoses could be confirmed in only 9% in normal hearts, the percentage increased to 17% in slight, to 25% in moderate, to 63% in severe, and to 95% in complete coronary stenosis. This series involved an impressive number of 403 autopsies. Fulton, who demonstrated the ubiquity of interarterial anastomoses in normal human hearts, also performed pathoanatomical investigations in CAD and nicely showed a right-shift of the collateral size distribution compared to controls [10, 16]. The relation between stenosis severity and angiographic collateral grade has been elegantly described in 36 CAD patients by Cohen *et al.* [44]. They furthermore demonstrated the collateral recruiting effect of brief coronary balloon occlusions. Piek *et al*. investigated additional clinical determinants of collateral formation in 58 patients referred to coronary angiography for 1-vessel disease who had not suffered from any previous myocardial infarction [45]. They found that in addition to stenosis severity, angina duration ≥ 3 months was also related to angiographic collateral grade. In another more sophisticated study, the same group determined clinical, angiographic and hemodynamic predictors of angiographic collateral grade in 105 patients with symptomatic, stable 1-vessel disease [46]. In multivariate analysis, the investigators found the following independent determinants of collateral formation: angina duration ≥ 3 months, diameter stenosis ≥ 75%, proximal lesion location, and absence of nitrate use. All four factors taken together seemed to indicate that collaterals tend to evolve along with ischemia severity and duration. In a larger study by Pohl *et al*. using quantitative collateral measurements in 450 CAD patients (50 of whom had previously suffered from myocardial infarction), the results of Piek *et al*. could partially be confirmed. In univariate analysis, the following variables were associated with CFI ≥ 0.25: percent diameter stenosis, number of vessels diseased, number of stenoses, area at risk, absence of previous Q-wave infarction, angina during treadmill exercise, presence and number of total chronic occlusions, other than left anterior descending artery (LAD) involvement, HDL cholesterol, and total/HDL cholesterol ratio. In multivariate analysis, however, percent diameter stenosis remained the only predictive factor for high CFI. 

In multivariate analyses involving CAD populations, the resulting predictors tend to account for a greater proportion of the observed CFI variability compared to non-CAD populations [30]. This demonstrates again the dominant role of CAD for collateral formation. But on the other hand, CAD might also cast a cloud over minor determinants. The non-appearance of ischemia-related factors in Pohl's multivariate analysis could be a question of statistical power, but it could also reflect the underlying physiological mechanisms. Collateral growth is mainly the result of a small vessel transformation into larger conductance vessels equipped with vasomotor capabilities [47, 48]. This remodeling process (arteriogenesis) is triggered by shear stress acting on endothelial cells [23], as opposed to the sprouting of new vessels (angiogenesis) induced by ischemic tissue. Angiogenesis seems to play a minor role in the myocardium. Considering that 1-vessel disease-patients were included in Piek's study [46], the pro-ischemic factors increased regional differences in peripheral vascular resistance and intercoronary pressure gradients rather than inducing collateral growth per se. Vasomotor processes involved in donor and receiver arteries have been elucidated by Billinger *et al.* [49]. In this context, it should also be mentioned that Billinger *et al*. demonstrated a collateral recruitment during repeated ischemic episodes, a mechanism that induced ischemia tolerance and that probably acted in concert with ischemic preconditioning at the cellular level [50, 51]. 

As for preformed collaterals, gene expression has also been investigated in the context of CAD-related collateral stimulation. Chittenden *et al*. investigated the transcriptional profile in circulating monocytes in 8 patients with angiographically poor, and in 8 patients with angiographically well-developed collaterals [52]. They identified a large number of markers of "noncollaterogenic" phenotype. They also suggested that by including these factors, the collateral formation is independent of the angiographic extent of CAD. It must be emphasized, however, that in the light of the 'noise' generated by gene expression analyses, serious concerns about the statistical power and reproducibility of this study are justified. In the previously mentioned study on gene expression by Meier *et al.*, 110 CAD patients were also included [21]. In this group, 8 marker genes were correlated significantly with CFI, and 3 were significant predictors of CFI ≥ 0.21. These were: DCP1A (tumor growth factor β pathway), MAP2K4, and PNPLA4. FGF13 (angiogenesis pathway) appeared in both, CAD and non-CAD group. In a more recent study in 226 CAD patients (84 with angiographically low, 142 with high grade collaterals), Zhang and Regieli analyzed 54 genetic variants in 41 inflammatory candidate genes [53]. They found that 16 genes were associated with high grade collaterals, and nine single nucleotide polymorphisms were potential determinants of collateral formation. Furthermore, several haplotype up- and down-interactions were identified at reasonable false discovery rates, being thus potentially involved in increased and decreased collateral formation, respectively. The importance of this study lies in the involvement of a series of pathways that are common to other clinical conditions, such as atherosclerosis, diabetes, stroke and others. The available data on clinical determinants of collateral (pre-) formation may help weighting the relevance of these pathways. 

In 42 patients with single-vessel CAD, Schirmer *et al*. analyzed the heterogeneity of transcriptional activity in circulating cells between “arteriogenic responders” (CFI>0.21) and “non-responders” (CFI≤0.21) matched for stenosis severity [54]. The cells analyzed included resting and stimulated monocytes, macrophages, and CD34+ progenitor cells. In stimulated monocytes, 95% of the 100 most differentially expressed genes were upregulated in non-responders. Pathway analyses revealed interferon-β (IFN-β) as an important upregulated pathway in non-responders. In subsequent experiments, IFN-β was shown to attenuate collateral formation in an established animal model, and to inhibit smooth muscle cell proliferation *in vitro*. This study not only documents a predominance of transcriptional cell-activity associated with poor collateral formation, but also strongly supports a causal relationship between IFN-β expression and poor collateralization. In the light of these results, inhibiting anti-arteriogenic rather than promoting pro-arteriogenic pathways seems a more promising therapeutic approach.

## SUMMARY

The determinants of coronary collaterals are still incompletely identified and understood. 

It seems that factors in early childhood play an important role for collateral preformation. The interarterial anastomoses spontaneously regress in size in a large proportion of individuals, but do not disappear completely. Collaterals are present in all humans, but they are functional only in about one fifth. Several clinical conditions in early childhood seem to counteract the natural regress of anastomoses, such as anemia. In adult life, a high heart rate and arterial hypertension appear detrimental for collateral function. However, 70% of the variability of collateral function has not yet been explained in individuals without CAD. The genetic predisposition may partly fill the gap. The strongest known stimulus for collateral formation is a coronary stenosis, with a direct relation between collateral formation and stenosis severity. In the shadow of CAD, it has been difficult to identify further determinants. Factors associated with severity of CAD in general, such as angina duration, number of diseased vessels and others have been proposed. Genes encoding pathways for angiogenesis and inflammatory processes have been related to collateral formation and deprivation. Clearly, the search for determinants of collaterals is not over. It will continue simply because the prospect of growing coronary bypasses is very attractive. 
